# Should pregnant women know their individual risk of future pelvic floor dysfunction? A qualitative study

**DOI:** 10.1186/s12884-022-04490-9

**Published:** 2022-02-28

**Authors:** Carol Bugge, Heather Strachan, Stewart Pringle, Suzanne Hagen, Helen Cheyne, Don Wilson

**Affiliations:** 1 Faculty of Health Sciences and Sport, University of Stirling, Stirling, FK9 4LA UK; 2 Obstetrics and Gynaecology, NHS Greater Glasgow and Clyde, Glasgow, UK; 3 Nursing, Midwifery and Allied Health Professions Research Unit, Glasgow Caledonian University, Glasgow, UK; 4 Nursing, Midwifery and Allied Health Professions Research, University of Stirling, Stirling, UK; 5 University of Otago, Christchurch, New Zealand

**Keywords:** Pelvic floor dysfunction, Urinary incontinence, Faecal incontinence, Pelvic organ prolapse, Risk, Maternity care

## Abstract

**Background:**

The study aimed to explore:

• pregnant women’s and healthcare professionals’ perspectives on provision of individual risk scores for future Pelvic Floor Dysfunction (PFD),

• the feasibility of providing this during routine maternity care,

• actions women might take as a result of knowing their PFD risk.

**Methods:**

Qualitative study. *Setting:* UK NHS Health Board. *Participants:* Pregnant women (*n* = 14), obstetricians (*n* = 6), midwives (*n* = 8) and physiotherapists (*n* = 3). A purposive sample of pregnant women and obstetric healthcare professionals were introduced to the UR-CHOICE calculator, which estimates a woman’s PFD risk, and were shown examples of low, medium and high-risk women. Data were collected in 2019 by semi-structured interview and focus group and analysed using the Framework Approach.

**Results:**

Women’s PFD knowledge was limited, meaning they were unlikely to raise PFD risk with healthcare professionals. Women believed it was important to know their individual PFD risk and that knowledge would motivate them to undertake preventative activities. Healthcare professionals believed it was important to discuss PFD risk, however limited time and concerns over increased caesarean section rates prevented this in all but high-risk women or those that expressed concerns.

**Conclusion:**

Women want to know their PFD risk. As part of an intervention based within a pregnant woman/ maternity healthcare professional consultation, the UR-CHOICE calculator could support discussion to consider preventative PFD activities and to enable women to be more prepared should PFD occur. A randomised controlled trial is needed to test the effectiveness of an intervention which includes the UR-CHOICE calculator in reducing PFD.

**Supplementary Information:**

The online version contains supplementary material available at 10.1186/s12884-022-04490-9.

## Background

Pelvic floor dysfunction (PFD) affects millions of women worldwide and is associated with considerable financial costs for healthcare systems and women. PFD adversely affects women’s well-being, quality of life, body image and sexual function [1–5]. The term Pelvic Floor Dysfunction incorporates three main conditions: urinary incontinence, faecal incontinence and pelvic organ prolapse. PFD risk factors have been identified as: greater parity, vaginal birth, advancing age, obesity, previous hysterectomy, and family history of PFD [6–9]. Interventions are available that may reduce risk of PFD development, such as weight reduction and pelvic floor muscle training (PFMT) [9–14], therefore early identification of women at high risk of PFD is important for its prevention [15]. Based on data from two large longitudinal cohort studies with long-term follow up, [6, 7, 16, 17] researchers have developed an online calculator (UR-CHOICE calculator), which estimates, during and beyond pregnancy, a woman’s individual risk of PFD in the long-term [15, 18]. Features about the woman (e.g. her weight), her pregnancy (e.g. the estimated size of her baby), and her birth history (e.g. mode of previous births) are all entered into the calculator and a set of individualised risk ‘scores’ are generated that predict probabilities of developing urinary incontinence, faecal incontinence, prolapse, and PFD overall. It is envisaged that the calculator will be used as part of a wider UR-CHOICE intervention, the additional components being identified through this qualitative study. However, the acceptability and feasibility of the UR-CHOICE intervention among women and maternity healthcare professionals (HCPs) has not been explored. This study therefore sought to answer the following research questions:

1. What are the perspectives of pregnant women on knowing their PFD risk while pregnant and what actions might they take if they know their risk?
2. What are maternity HCPs views about delivering the UR-CHOICE intervention to a woman during routine antenatal care and in the postnatal period and on engaging in discussion about PFD risk?

## Methods

The study used qualitative methods. Data were collected (by authors HS and CB) using semi-structured telephone interviews with pregnant women, obstetricians and physiotherapists and a focus group with midwives. The different data collection approaches were selected to maximise the richness of the data while ensuring approaches that were appropriate to the different participant groups [19]. Different methods were used to support data saturation and collection of a large volume of data in a short time [20]. For example, as the study focussed on a sensitive topic individual interviews were chosen for women which generated in-depth data at an individual level, while focus groups were chosen for midwives as it was feasible to gather a group at one point in time and it gave an opportunity for professional discussion to generate a range of viewpoints. Data analysis used the Framework Approach [21]. Funding was from the Chief Scientist Office, Scottish Government (CGA/18/39). The application was peer reviewed for scientific quality and the funders had no role in undertaking the research or in manuscript preparation. A steering group, which included a pregnant woman, oversaw the design, conduct and writing up of the research. Ethical approval was granted by London-Brighton and Sussex Research NHS Research Ethics Committee (19/LO/0527) on 12th April 2019.

### Setting and context

The study was undertaken in the antenatal clinics of a large teaching hospital in a Scottish NHS Health Board serving an urban population of approximately 1.2 million people. There are 5500 deliveries annually within the Board area with a 35% Caesarean Section rate. The Board have 34% of clients living in areas of high deprivation.

### Sample and recruitment

Purposive sampling of pregnant women aimed to recruit 15 women with diversity in parity (a mixture of primigravid and multiparous women), maternal age, and socioeconomic group. Inclusion criteria were women who attended ante-natal clinics, were pregnant and aged 16 years or older. Exclusion criteria were women without capacity to consent, or unable to understand English sufficiently to provide consent.

To achieve the required diverse and purposive sample initially midwives were asked to give women a study recruitment pack (an introductory letter, Participant Information Leaflet, permission to contact reply slip, consent form and stamped addressed envelope) as part of information given at booking. However, this recruitment strategy was unsuccessful, possibly because of the large amount of information women receive at booking. Therefore, following ethical and management approval, recruitment was extended to all antenatal clinics and antenatal classes. Midwives were asked to introduce the study to eligible women during their antenatal appointment and hand the woman a recruitment pack. A researcher (HS) was available at some antenatal clinics and antenatal classes to speak with women, explain the study and seek permission to contact them, or women could take the pack, read it, and return the reply slip if they wished to participate. Women who gave their permission to be contacted were telephoned to discuss the study and have their questions answered. An appointment was made for a telephone interview with those women willing to take part. Written consent was obtained prior to interview.

Community and hospital-based midwives were sent an email invitation containing a Participant Information Leaflet, consent form, cascaded via the Lead Midwife. The aim was to achieve a purposive sample of midwives from different clinical areas. Written consent to participate was obtained from the midwives prior to the focus group.

Specialist Registrar and Consultant Obstetricians were invited to participate via email, containing an invitation letter, a Participant Information Leaflet and consent form, from the site Principal Investigator (a Consultant Obstetrician). The aim was to achieve a purposive sample of obstetricians with varying levels of experience. Those obstetricians interested in participating contacted the researchers who telephoned them to discuss the study and arrange a date for the telephone interview with written consent obtained prior to interview.

Physiotherapists providing services to pregnant women, were invited to participate in the study similarly to the obstetricians, via the Lead Physiotherapist, with a view to a purposive sample of varying levels of experience in women’s health.

Overall, the study aimed to recruit a maximum variation sample of pregnant women and HCPs to identify themes that cut across the variance to support implementation of a possible clinical trial [22]. A sample of 15 women was proposed to support the achievement of saturation in women’s perspectives where the women would have a variety of characteristics [23]. For example, the different risks of primigravida and multiparous women offers data from the widest, and hence most pragmatic perspective, critical to a potential pragmatic trial. The additional perspectives of 15 multi-disciplinary HCPs aimed to achieve saturation and maximum variation perspectives into the practical clinical world and possibilities of delivering the UR-CHOICE intervention as part of care delivery to pregnant women.

### Data collection

The interview schedule for women was developed based on guidance for the development of complex interventions [24]. The questions focussed on areas of acceptability of the intervention and its context. Women were therefore asked about: current knowledge of PFD, views on knowing their PFD risk and they were introduced to the UR-CHOICE calculator (Additional file 1). Women were not given their own risk scores, they were shown examples for low, medium and high-risk women (Additional file 2). They were asked how they would feel about engaging with the UR-CHOICE intervention with a HCP. The UR-CHOICE calculator was able to identify women at risk approximately 65% of the time in a validation dataset [15]. It is currently the best predictive tool available for PFD with a concordance index equivalent to other predictive tools currently in use in the NHS in the UK; for example, the National Cancer Institute Gail Model for prediction of Breast Cancer Risk [25] and the Framingham Cardiovascular risk model [26]. In the interview women were told that the figures were an estimation based on two large studies of women over 12 and 20 years. If participants raised concerns about their personal risk of PFD, they were directed to the information in their antenatal book and their maternity care team.

HCPs were asked, in focus group or interview (Additional file 3), about the same topics as were covered with the women. Specifically, their current practice in relation to PFD, their views on discussing risk of PFD with women during pregnancy and in the post-partum period. Examples of UR-CHOICE risk calculations for low, medium and high-risk women were demonstrated (Additional file 2) and opinions were sought on whether and how they might deliver the UR-CHOICE intervention in practice.

All interviews and the focus group were digitally recorded, transcribed verbatim and anonymised.

### Data analysis

The anonymised transcriptions were transferred into NVIVO 12 software and analysed using the Framework Approach [21]. Framework analysis is a flexible, systematic, and rigorous method of analysis that provides transparency and an audit trail. It facilitates easy retrieval of coded data and analysis to be undertaken on a case by case basis and in a theme-based way using a mixture of deductive coding based on the initial thematic framework and an inductive, open-coding approach based on additional emerging themes agreed and regularly reviewed by two researchers for data saturation (HS, CB). In this study the process involved:

- *familiarisation* by reading transcripts and documenting memos;
- constructing an *initial thematic framework* by drawing upon ideas identified through familiarisation, the research questions and the interview/focus group schedules;
- *indexing* by applying the thematic framework to the data;
- *reviewing the data extracts* by looking at data within a code and considering alternative ways of coding and organising the data;
- *summarising data and displaying it* into framework matrices for each major theme and on a case basis
- *abstraction and interpretation of the data* by reviewing the matrices, developing categories that represent the key features of the data and how they connect to one another.

## Results

### Characteristics of the sample

Recruitment took place between May and August 2019. Pregnant women (*n* = 14), midwives (*n* = 8), obstetricians (*n* = 6), and physiotherapists (*n* = 3) were recruited. Recruited pregnant women were aged 25–38 years; seven women were primigravidae and seven were multiparous, having their second (*n* = 5) or third child (*n* = 2). Five women lived in the most deprived quintiles [1 or 2] and nine lived in the most affluent quintiles [4 or 5] based on the Scottish Index of Multiple Deprivation [27].

Obstetricians included Consultants (*n* = 4) or Specialist Registrars (*n* = 2) with 7–30 years’ experience. Four worked across obstetrics and gynaecology. The midwives (*n* = 8) worked as part of an integrated maternity service across all specialties. Three physiotherapists had 20–28 years obstetric experience.

### Key themes

A number of inter-related themes were identified about current PFD practice, views on women knowing their PFD risk and on the UR-CHOICE intervention.

#### PFD risk is an important yet un-broached health issue

All women interviewed wanted to know their risk of developing PFD. They believed it was important information that would allow them to make informed decisions about prevention and treatment or be prepared to manage the problem if likely or inevitable.

*“It (knowing PFD risk) would be quite helpful because it means that you could prepare and it means that if you know what the risks are, is there anything that you can do to prevent it.”*

*P08 (woman),*

Potential reactions to discussing PFD risk highlighted by women and HCPs included anxiety, worry or embarrassment and the addition of another potential problem in pregnancy. However, despite this, women still believed that it was better to know than not.

*“..it’s (being high risk of PFD) not something, I suppose, you want to hear about, but … you’d rather know, than not know. As long as there was something, I could do about it.”*

*P012 (woman)*

Women believed that if the HCP opened the dialogue about PFD it would help them talk about it. Various justifications included: legitimising the problem and its importance, helping them feel supported or less embarrassed and encouraging them to seek help if needed.

*“doing it (discussing PFD risk) with a health professional adds that kind of understanding of the risk and it makes it more serious I suppose rather than, you know, if you’re just given a leaflet or something like that.”*

*P011 (woman)*

#### There is a tension between women-driven care and their limited PFD knowledge

Information about pelvic floor health was included in a book given to women at their booking appointment, but women reported that they did not read it. Unless women had attended antenatal classes their knowledge about PFD, the risks, prevention and treatment was limited. The limited knowledge they had was gained from family and friends. Whilst, women with PFD symptoms could self-refer to a physiotherapist this relied on women being signposted by an HCP or obtaining information about self-referral procedures from posters in antenatal clinics. Additionally, women perceived urinary incontinence was an inevitable consequence of having children and something “just to be put up with”. Consequently, if neither women or HCPs raised PFD it was not discussed and women were unlikely to gain more knowledge about PFD or its prevention. HCPs and women believed this was compounded by urinary incontinence product adverts that normalised urinary incontinence.

*“And then the next advert comes on, and it's somebody going to a wedding, and she's pulling on incontinence pads. So that’s saying, it's alright to be peeing yourself.”*

*P01 (midwife)*

#### Current maternity healthcare PFD practice is problem orientated rather than prevention orientated

HCPs do not routinely discuss PFD with all women during the antenatal or postnatal period. The exception was antenatal classes but not all women attend these. Obstetricians only ask women about PFD symptoms if they have a history of 3rd/4th degree tear. Postnatally, women who have had an assisted delivery or 3rd/4th degree tear were seen by a physiotherapist and referred for treatment. Women may or may not raise the issue of PFD symptoms with their HCP during their pregnancy. If women report PFD symptoms these are discussed, or they may be signposted to a specialist physiotherapist.

*"I suppose antenatally … it’s not one of our routine things that we ask. If people sometimes bring it up we always recommend that they do their pelvic floor exercises, but we don’t have a kind of formal referral pathway or formal time that we ask people about it”."*

*P01 (Doctor)*

Despite not raising the issue of PFD routinely, HCPs believed discussing PFD risk and prevention strategies was important and they were “duty bound’ to tell women,

*“We shouldn’t be doing anything to a patient that is not without their full knowledge, but the problem with that is that a lot of the stuff (information) is complicated … … when patients are dealing with a huge amount and huge wealth of information, I think it can be a very difficult thing for patients to fully understand and to not worry about the minutiae of the risks of things to happen to them”*

*P03 (Doctor)*

HCPs recognised women’s right to choose their preferred mode of birth but were concerned women with a high PFD risk score would choose caesarean section, which itself had risks, believing women perceived a caesarean section to be more protective than it is in reality. Whilst this perception was confirmed by some women, most women did not mention choosing caesarean section as a preventative strategy despite being shown the different risks associated with each birth mode. Some women reported that the intervention had been informative in terms of highlighting risk associated with both pregnancy and birth.

*“See, I would have thought probably labour, mainly. I’m now kind of more aware that actually the pregnancy itself can. But in my head, beforehand, I know that before I got pregnant, I would always just kind of think it was more the labour that would then cause these kinds of problems.”*

*P04 (Woman)*

#### Lower priority in a time constrained service created barriers to discussing PFD risk

Despite articulating the importance of discussing PFD risk, HCPs’ main focus was on the safe birth of the baby and immediacy of pregnancy and labour rather than long-term health issues for women. There was limited time to discuss PFD risk and prevention activities with women during HCP consultations. HCPs also believed women may have information overload during their antenatal and postnatal care or may not understand the complexity of risk.

*“it’s an important aspect for women to understand, what vaginal delivery can do to the pelvic floor and what they should be doing afterwards, but I would say it’s a time constraint mainly, and a lot of other things we want to finish in that 10-15 minute consultation, baby chat and all that, but yeah, it kind of sneaks down to the bottom of the list”*

*P02 (Doctor)*

#### Views on the UR-CHOICE intervention suggested the importance of promoting informed choice whilst recognising complexity of different risks

Participants suggested that the UR-CHOICE intervention should include a wider ranging discussion alongside using the UR-CHOICE calculator to identify the individual woman’s PFD risks. They proposed: information provision about the woman’s risk factors and how a woman’s pelvic floor was affected by these; preferred preventative and treatment options and their attendant risks for the woman, their baby and future family planning; a care plan for PFD prevention or treatment agreed upon by the woman and HCP.

The study participants reported that the UR-CHOICE intervention was acceptable and could be delivered second trimester, late pregnancy and postnatally. A range of professionals were viewed as appropriate to deliver the UR-CHOICE intervention including midwives, physiotherapists, obstetricians, advanced nurse practitioners or practice nurses in primary care. Women believed midwives were the most appropriate HCP, whilst midwives and physiotherapists believed specialist physiotherapists had the most appropriate expertise to discuss PFD with women. Doctors believed midwives were best placed due to the rapport they had with women and because obstetricians and physiotherapists did not see all women.

#### Perceived benefits of, and concerns about, UR-CHOICE

Participants reported that the UR-CHOICE intervention could increase women’s knowledge of PFD, facilitate informed decision-making and motivate women to engage in PFD prevention. Some women believed there were no drawbacks to UR-CHOICE, whilst some women did not know their family history and did not feel able to discuss PFD with their mothers and as such felt they might not be able to provide the information required for the calculator. Concerns from HCPs included potential increases to caesarean section rates and consultation times. There were also concerns that women identified as low risk might not engage in preventative PFD activities.

## Discussion

### Main findings

Current PFD practice in maternity care was orientated towards PFD problems rather than prevention. Practice focussed on women-led care, but women lacked knowledge about PFD so may not raise or identify problems. All HCPs recognised PFD was an important issue for pregnant women, and all women indicated that they wanted to know their risk. However, time constraints on the service and the immediate safety of mother and baby meant PFD was not routinely discussed. HCPs were concerned that discussing PFD risk could increase caesarean section rates. Women and HCPs reported that implementation of the UR-CHOICE intervention was feasible and acceptable.

### Strengths and limitations

This study sought women’s views about knowing their potential PFD risk, alongside those of HCPs, and in doing so supports service development in women-centred ways. The study was undertaken in one regional UK hospital and therefore may not represent cultural issues or current practice in other regions or countries. Recruitment of pregnant women was challenging due to busyness of clinics and the final sample did not cover the entire age range of pregnant women as it did not include those under 24 years and data on ethnicity was not gathered. It is possible that the women who agreed to take part had a greater interest in PFD prevention than those who did not. However, the sample did achieve variance with women who had a range of pregnancy and socio-demographic characteristics, although no sub analysis was conducted due to the small sample. The sample included midwives, women’s health physiotherapists and obstetricians with a range of experience; in this way a range of HCP views were achieved. By nature of the qualitative study design, the sample was varied, but may not represent the views of a wider group of pregnant women or HCPs. To move forwards, further consultation with pregnant women and midwives would be essential to maximise the best trial recruitment pathways.

The interview did not include discussion of the exact calculator concordance index, but women were advised that the figures were an estimation based on two large studies over time. It is possible that if women knew the exact index they would have felt less positive about knowing PFD risk. However, the concordance index of UR-CHOICE is in keeping with other risk tools that are routinely used within the NHS [25, 25]. Given the pattern of evidence in this study and elsewhere [28] that women do want to know and understand risk as part of their healthcare, offering risk information to women seems important. However, exploring women’s views about the accuracy of the prediction and how that would affect their actions will be important to consider as part of a larger trial.

The UR-CHOICE calculator has some current limitations, for example it only includes an age range up to 34 years. However, the calculator will be updated prior to further research based on further data from the longitudinal studies that underpin it.

### Rigour

Rigour is demonstrated using Ballies (2015) four categories of trustworthiness: transferability, credibility, dependability, and confirmability [29]. The study demonstrated transferability by reaching data saturation; credibility by analysing verbatim transcripts and using quotes to demonstrate conclusions; dependability by exploring the same topics with all participants and confirmability by asking HCP to review findings.

### Interpretation

PFD is common [16, 17], adversely impacts women’s wellbeing [1, 3], and has a major economic burden for women and healthcare organisations [30, 31]. Therefore, PFD prevention is important for women and services alike. Whilst directing resources to those at highest risk and to immediate safety requirements may be understandable in resource-constrained services, PFD prevention was deemed clinically important to women and HCPs. Similar to a Canadian study, PFD information was lacking in antenatal practice and HCPs were concerned about increased caesarean section rates but concerns about negatively influencing women’s preference for mode of delivery were unwarranted [32]. As the UR-CHOICE intervention is potentially feasible and acceptable to those who would deliver and receive it, it offers a potential structure for managing PFD information in practice.

Informed consent and personal control principles are fundamental to high quality maternity care. Recent reports have highlighted high profile failures to respect these principles [33, 34]. There is now evidence, based on large cohort studies and reviews of evidence, that offers insight into the risk factors for PFD for women [9, 16, 17] that has been used to develop a calculator which can offer women information about their individual risk [15, 18] and has a concordance index that is equivalent to, or better than, other predictive tools in use in the UK. Given the accessibility of this evidence via the UR-CHOICE intervention it has been prudent to assess the usefulness of delivering that evidence to women in practice.

PFD may not be wholly preventable, consequently talking about it may raise anxiety, however, there are interventions that can reduce PFD symptoms and severity [9–14, 28] for example, PFMT [11, 14]. Perhaps more importantly, in this study women recognised that knowing their PFD risk may raise anxiety but their unanimous preference was to know. Although concerned about the complexity of PFD risk information for women, HCPs already discuss complex risks and options related to conditions such as Down’s Syndrome, and to delivery options such as caesarean section [35]. When discussing risk women wanted to be informed about lowering their risk of PFD occurrence. One proposed mechanism of action of the UR-CHOICE intervention is that if women know their risk, they may be more motivated to undertake preventative/alleviative activities which in turn may decrease the prevalence and morbidity associated with PFD. The evidence reported here supports that mechanism of action.

In developing the UR-CHOICE calculator, it was envisaged that it could be used as part of a woman-HCP discussion. The findings have identified components of, and structure for, that discussion that may support women having full information about their PFD risks, alongside other risks (such as risks of caesarean section). The second mechanism of action for the UR-CHOICE intervention is that decisions about the woman’s birth (made by women and HCPs) are informed by knowledge of her PFD risk in a way that prevents/reduces PFD, such as choosing the most appropriate mode of delivery or influencing other lifestyle factors. The evidence presented here offers some structure for how the intervention may be developed to support a holistic, woman-focussed approach to that mechanism of action.

There was discrepancy in the views about who would best deliver UR-CHOICE in practice. Women and obstetricians reported midwives as the most appropriate HCP to deliver the UR-CHOICE intervention, however midwives identified physiotherapists. This disparity could be related to midwives lacking confidence in their ability to talk about and deliver pelvic health interventions, such as PFMT, in practice [36] or to the additional workload this may create.

## Conclusion

### Implication for research and practice

There is evidence to support further development and testing of the UR-CHOICE intervention in practice. The study has identified three elements to the UR-CHOICE intervention: risk assessment (the calculator); discussion of preventative/alleviative strategies and a care plan. The discursive elements of the package and who will deliver it need further consideration and a clear clinical pathway documented.

The clinical pathway could include preventative and treatment options that can be performed by HCPs, the women independently, or in partnership. UR-CHOICE has a focus on pregnancy and the post-natal period, but interventions may also be incorporated into public health practice. Already women are directed to undergo various screening activities such as cervical smear test in the UK. UR-CHOICE could be introduced alongside these other public health interventions, utilising other HCPs, perhaps in pelvic health clinics [37].

The UR-CHOICE intervention has the potential to support HCPs to provide personalised health care that involves pregnant women in decisions by providing knowledge about *their* individual PFD risk and motivating them to reduce the likelihood of PFD. Increasing preventative measures associated with PFD could lessen the incidence of, or morbidity associated with, a major health issue that affects approximately 50% of women. Ultimately, this potential needs tested within a randomised controlled trial.

## Supplementary Information

**Additional file 1.** Interview Schedule for Pregnant Women Participants. The interview schedule used with pregnant women in the study.

**Additional file 2.** URCHOICE calculator example results shown to healthcare professionals and women (high risk and low risk only) participants at interview/focus groups. The examples of the URCHOICE calculator that were used during interviews with pregnant women and health care professionals.

**Additional file 3.** Interview Schedule for Healthcare Professionals. The interview schedule used with health care professionals in the study.

## Data Availability

The datasets generated and/or analysed during the current study are not publicly available as participant consent was not given for data sharing beyond the research team but may be available from the corresponding author on reasonable request and if ethical permissions are given.

## Abbreviations

HCP: Health Care Professional
PFD: Pelvic Floor Dysfunction
PFMT: Pelvic Floor Muscle Training

## References

[1] Toye F, Pearl J, Vincent K, Barker K. A qualitative evidence synthesis using meta-ethnography to understand the experience of living with pelvic organ prolapse. IUJ. 2020. 10.1007/s00192-020-04494-z. 10.1007/s00192-020-04494-z PMC7459259 32870341

[2] Hamid TA, Pakgohar M, Ibrahim R, Dastjerdi MV (2015). "Stain in life": The meaning of urinary incontinence in the context of Muslim postmenopausal women through hermeneutic phenomenology. Arch Gerontol Geriatr.

[3] Coyne K, Sexton C, Irwin D, Kopp Z, Kelleher C, Milsom I (2008). The impact of overactive bladder, incontinence and other lower urinary tract symptoms on quality of life, work productivity, sexuality and emotional well-being in men and women: results from the EPIC study. BJU Int.

[4] Jelovsek JE, Barber MD (2006). Women seeking treatment for advanced pelvic organ prolapse have decreased body image and quality of life. Am J Obstet Gynecol.

[5] Kenton K, Mueller ER (2006). The global burden of female pelvic floor disorders. BJU Int.

[6] Gyhagen M, Bullarbo M, Nielsen TF, Milsom I (2013). The prevalence of urinary incontinence 20 years after childbirth: a national cohort study in singleton primiparae after vaginal or caesarean delivery. BJOG.

[7] Gyhagen M, Bullarbo M, Nielsen TF, Milsom I (2013). Prevalence and risk factors for pelvic organ prolapse 20 years after childbirth: a national cohort study in singleton primiparae after vaginal or caesarean delivery. BJOG.

[8] Barber M (2016). Pelvic organ prolapse. BMJ.

[9] Abrams P, Cardozo L, Wagg A, Wein A (2017). Incontinence 6th Edition.

[10] National Institute of Clinical Excellence (2019). Urinary continence and pelvic organ prolapse in women: management.

[11] Dumoulin C, Cacciari LP, Hay-Smith EJC. Pelvic floor muscle training versus no treatment, or inactive control treatments, for urinary incontinence in women. Cochrane Database of Syst Rev. 2018;(10):CD005654. 10.1002/14651858.CD005654.pub4. Accessed 25 Feb 2022. 10.1002/14651858.CD005654.pub4 PMC6516955 30288727

[12] Hagen S, Glazener C, McClurg D, Macarthur C, Elders A, Herbison P (2017). Pelvic floor muscle training for secondary prevention of pelvic organ prolapse (PREVPROL): a multicentre randomised controlled trial. Lancet.

[13] Bugge C, Adams EJ, Gopinath D, Stewart F, Dembinsky M, Sobiesuo P, et al. Pessaries (mechanical devices) for managing pelvic organ prolapse in women. Cochrane Database Syst Rev. 2020, Issue 11. Art. No.: CD004010. 10.1002/14651858.CD004010.pub4. 10.1002/14651858.CD004010.pub4 PMC8094172 33207004

[14] Hagen S, Stark D (2011). Conservative prevention and management of pelvic organ prolapse in women. Cochrane Database Syst Rev.

[15] Jelovsek J, Chagin K, Gyhagen M, Hagen S, Wilson D, Kattan M (2017). Predicting risk of pelvic floor disorders 12-20 years after delivery. AJOG.

[16] Gyhagen M, Åkervall S, Milsom I (2015). Clustering of pelvic floor disorders 20 years after one vaginal or one cesarean birth. Int Urogynecol J.

[17] MacArthur C, Wilson D, Herbison P, Lancashire RJ, Hagen S, Toozs-Hobson P, Dean N, Glazener C (2016). Prolong study group. Urinary incontinence persisting after childbirth: extent, delivery history, and effects in a 12-year longitudinal cohort study. BJOG.

[18] Wilson D, Dornan J, Milsom I, Freeman R (2014). UR-CHOICE: can we provide mothers-to-be with information about the risk of future pelvic floor dysfunction?. Int Urogynecol J.

[19] Lambert SD, Loiselle CG (2008). Combining individual interviews and focus groups to enhance data richness. J Adv Nurs.

[20] Fusch P, Ness L (2015). Are we there yet? Data saturation in qualitative research. Qual Rep.

[21] Ritchie J, Lewis J, Nicholls CM, Ormston R (2014). Qualitative research practice.

[22] Patton MQ (2014). Qualitative research and evaluation methods.

[23] Guest G, Bunce A, Johnson L (2006). How many interviews are enough?: an experiment with data saturation and variability. Field Methods.

[24] MRC (2008). Developing and evaluating complex interventions: new guidance.

[25] Rockhill B, Spiegelman D, Byrne C, Hunter DJ, Colditz GA (2001). Validation of the Gail model of breast cancer risk prediction and implications for chemoprevention. J Natl Cancer Inst.

[26] SR D’ARB, Grundy S, Sullivan LM, Wilson P, Group CHDRP (2001). Validation of the Framingham coronary heart disease prediction scores: results of a multiple ethnic groups investigation. JAMA.

[27] Scottish Government (2020). Scottish Index of Multiple Deprivation 2020.

[28] Independent Medicines and Medical Devices Safety Review (IMMDS) (2020). First do not harm – The report of the IMMDS review [led by Baroness Cumberlege].

[29] Baillie L (2015). Promoting and evaluating scientific rigour in qualitative research. Nurs Stand.

[30] Coyne KS, Wein A, Nicholson S, Kvasz M, Chen CI, Milsom I (2014). Economic burden of urgency urinary incontinence in the United States: a systematic review. J Manag Care Pharm.

[31] Turner D (2004). The costs of stress urinary incontinence. Health Serv J.

[32] Hyakutake MT, Han V, Cundiff GW, Baerg L, Koenig NA, Lee T, Geoffrion R (2016). Pelvic floor health education: can a workshop enhance patient counseling during pregnancy?. Female Pelvic Med Reconstr Surg.

[33] Judgment Montgomery (Appellent) vs Lanarkshire Health Board (Respondent) (Scotland). 2015. Available from: https://www.supremecourt.uk/cases/docs/uksc-2013-0136-judgment.pdf. [cited 2018 July 24].

[34] Glazener C, Breeman S, Elders A, Hemming C, Cooper K, Freemen R (2017). Mesh, graft, or standard repair for women having praimary transvaginal anterior or posterior compartment prolapse surgery: two parallel-group, multicenter, randomized, controlled trials (PROSPECT). Lancet.

[35] National Health Service (2019) (2019). Caesarean Section.

[36] Perry S, Shaw C, McGrother C, Matthews RJ, Assassa RP, Dallosso H (2002). Leicestershire MRC Incontinence Study Team. Prevalence of faecal incontinence in adults aged 40 years or more living in the community. Gut.

[37] National Health Service (2019) The NHS Long Term Plan. Available from https://www.longtermplan.nhs.uk/publication/nhs-long-term-plan/. [accessed 11.03.21].

