# Supplementary material for: Should pregnant women know their individual risk of future pelvic floor dysfunction? A qualitative study
Source: BMC Pregnancy Childbirth. 2022 Feb 28;22:161. doi: 10.1186/s12884-022-04490-9 (PMC8883628; doi:10.1186/s12884-022-04490-9)
Supplement: Supplementary file 1 — Additional file 1. Interview Schedule for Pregnant Women Participants. The interview schedule used with pregnant women in the study. [file 12884_2022_4490_MOESM1_ESM.docx]

**Additional File 1**

**Interview Schedule for Pregnant Women Participants**

***Investigating pregnant women’s and health care professional’s view about knowing a woman’s individual risk of future pelvic floor dysfunction: a feasibility study for the UR-CHOICE Randomised Controlled Trial***

## Introduction to study and self

Thank you for agreeing to be interviewed. We greatly appreciate your willingness to help with the UR-CHOICE interview study. The UR-CHOICE study aims to find out about what women think about knowing their risk of pelvic floor dysfunction during pregnancy and after their baby is born. I am [introduce researcher undertaking interview], one of the researchers on the UR-CHOICE study.

## Consent

Go over study and what is involved. Particularly emphasise following points. There are no right or wrong answers, we are interested in your views. All data will be kept confidential. If at any time you wish to stop the interview you can do so, you do not need to give a reason. Do you have any questions for me? Are you still happy to be interviewed and for that interview to be recorded? Check signed consent for has been returned.

## Introduction to interview

Today’s interview is about your views about pelvic floor dysfunction and some research we plan to undertake in the future. It will take approximately 60 minutes.

## Ice breaker

- How has your health been during your pregnancy?
- Can you tell me a little about yourself (age, previous pregnancies, how many weeks pregnant with this pregnancy)

## Knowledge of PFD

- Do you know anything about pelvic floor dysfunction?
- What do you think it is?
  - Probe about UI, FI and prolapse
  - [If woman knows nothing about PFD a brief explanation of the main symptoms – UI, FI, prolapse – will be given].
- Any PFD symptoms in any previous pregnancies or more generally?
- Has anyone discussed PFD with you this pregnancy?
- Explore PFD knowledge from family and friends with experience

## View on knowing PFD risk while pregnant

- What are your thoughts about discussing your PFD risk with a health professional while you are pregnant and after the birth of your baby?
- What would you want to know and why?
- What impact do you think having that information would have for you?
  - Benefits
  - Drawbacks
- If your risk was high or very high what do you think you would do? How do you think you would feel?
- Do you know of any actions women could take to reduce their risk:
  - Probe PFMT
  - Probe lifestyle factors (maintaining normal weight)

## Introduction to UR-CHOICE process

- Show woman the calculator examples – completed for fictional woman (one high risk and one low risk). Talk to her about what she sees.
- How would you feel if this was completed for you with your health care professional?
- What would you want to know?
- When would be the best time to do this (if at all)
- Who would you like to talk about this with (if anyone)
  - Probe obstetrician
  - Probe midwife
  - Probe primary care
- Do you think knowing this kind of information would make you feel anxious?
- Would you do something about it to help reduce likelihood of it happening if you knew this kind of information?
- If you were to be offered an opportunity to be in a trial about the UR-CHOICE process – do you think that you would (hypothetically) be willing or not?

## Closure

Thank you for talking with me today. The interview we have recorded will be removed from the recording device as soon as possible and stored securely. When the interview is typed up (transcribed), all identifying information will be removed. We will study the information you have given us alongside that given by other women. It will not be possible to identify you from the information given.

## Desire to have study results sent to her

Explore if woman would like to have a summary of the study results sent to her and if so if it is acceptable to send them to her at the address provided on the reply slip.

## Thank you for taking part.
