# Supplementary material for: Should pregnant women know their individual risk of future pelvic floor dysfunction? A qualitative study
Source: BMC Pregnancy Childbirth. 2022 Feb 28;22:161. doi: 10.1186/s12884-022-04490-9 (PMC8883628; doi:10.1186/s12884-022-04490-9)
Supplement: Supplementary file 2 — Additional file 2. URCHOICE calculator example results shown to healthcare professionals and women (high risk and low risk only) participants at interview/focus groups. The examples of the URCHOICE calculator that were used during interviews with pregnant women and health care professionals. [file 12884_2022_4490_MOESM2_ESM.docx]

**Additional File 2**

**URCHOICE calculator example results shown to healthcare professionals and women (high risk and low risk only) participants at interview/focus groups**

**Case 1: low risk primigravida**

- 28 year old
- Primigravid woman
- 150 pounds (68 kg), 5 feet 4 inches (162 cm)
- Estimated Fetal Weight = 7 pounds 2 ounces (3232 g)
- Fetal Head Circumference = 35 cm
- No history of diabetes
- No history of Urinary Incontinence before or during pregnancy
- No family history of Pelvic Organ Prolapse, Urinary Incontinence or Faecal Incontinence

**Results**


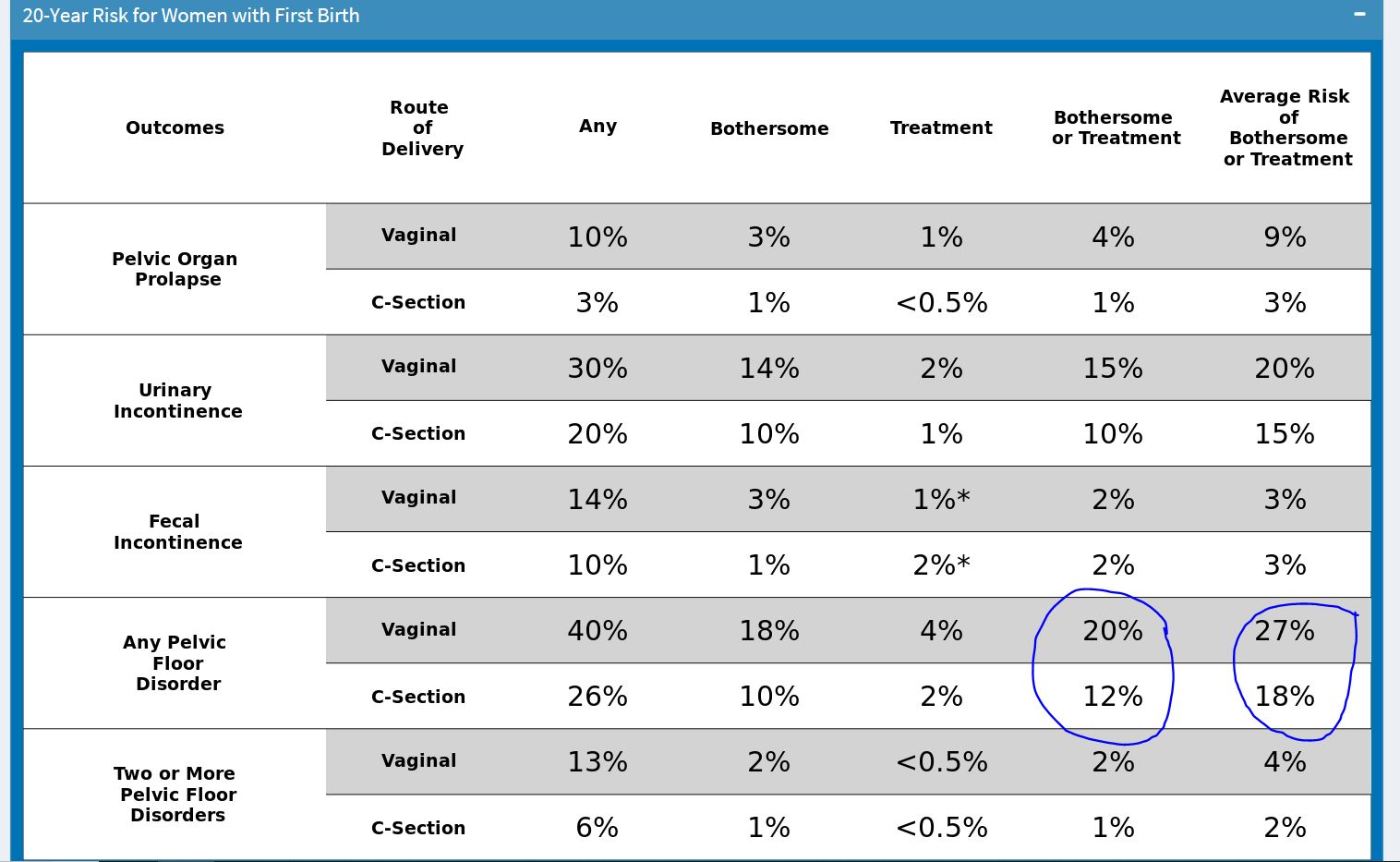


**Case 2: medium risk primigravida**

- 28 year old
- Primigravid woman
- 150 pounds (68 kg), 5 feet 4 inches (162 cm)
- Estimated Fetal Weight = 7 pounds 2 ounces
- Fetal Head Circumference = 35 cm
- Positive history of diabetes
- Urinary Incontinence before pregnancy
- Positive family history of Pelvic Organ Prolapse and Urinary Incontinence

**Results**


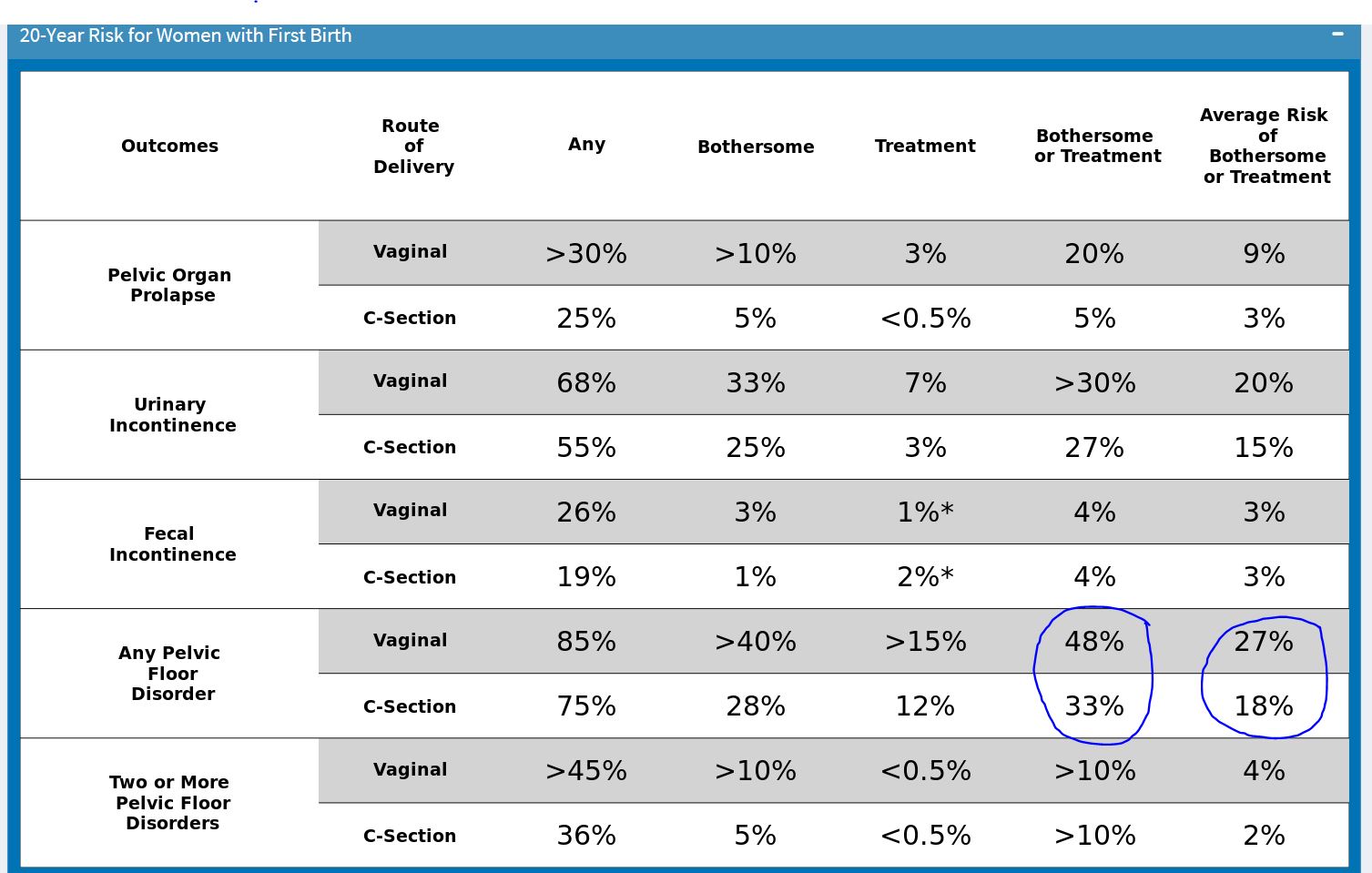


**Case 3: high risk multigravida**

- 30 year old
- 3^rd^ baby
- 1^st^ baby assisted vaginal delivery (forceps), 2^nd^ baby unassisted vaginal delivery
- 160 pounds (72 kg), 5 feet 8 inches (173 cm)
- Estimated Fetal Weight = 6 pounds 8 ounces (2948 g)
- Fetal Head Circumference = 32 cm
- No history of diabetes
- Urinary Incontinence pre and during pregnancy
- Family history of Urinary Incontinence

**Results**

**
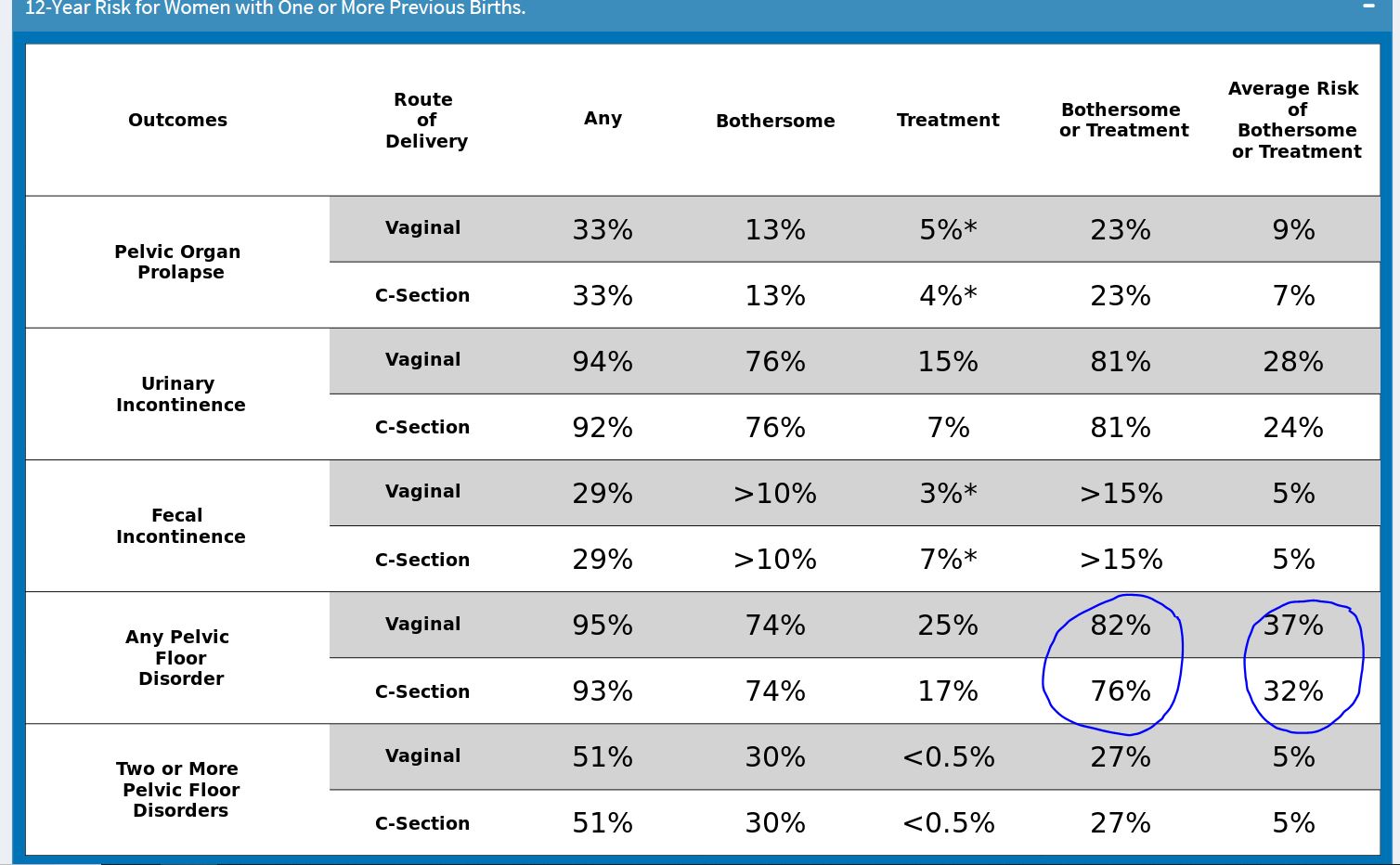
**
