# Supplementary material for: Should pregnant women know their individual risk of future pelvic floor dysfunction? A qualitative study
Source: BMC Pregnancy Childbirth. 2022 Feb 28;22:161. doi: 10.1186/s12884-022-04490-9 (PMC8883628; doi:10.1186/s12884-022-04490-9)
Supplement: Supplementary file 3 — Additional file 3. Interview Schedule for Healthcare Professionals. The interview schedule used with health care professionals in the study. [file 12884_2022_4490_MOESM3_ESM.docx]

**Additional File 3**

**Interview Schedule for Healthcare Professionals**

***Investigating pregnant women’s and health care professional’s view about knowing a woman’s individual risk of future pelvic floor dysfunction: a feasibility study for the UR-CHOICE Randomised Controlled Trial***

## Introduction to study and self

Thank you for agreeing to be interviewed. We greatly appreciate your willingness to help with the UR-CHOICE interview study. The UR-CHOICE study aims to find out about what women and health professionals think about knowing their risk of pelvic floor dysfunction during pregnancy and after their baby is born. I am xxx, one of the researchers on the UR-CHOICE study.

## Consent

Go over study and what is involved. Stress confidential nature of study. Do you have any questions for me? Are you still happy to be interviewed and for that interview to be recorded? **Check consent has been received.**

## Introduction to interview

Today’s interview is about your views about pelvic floor dysfunction and some research we plan to undertake in the future. It will take approximately 30 minutes.

## Ice breaker

- How long have you worked in Obs and Gyn ?
- Can you tell me a little about your background in Obs and Gyn
- How long in current post?

## Knowledge and practice about PFD

- What is your current practice in relation to PFD with pregnant women?
- Do you currently talk to women about PFD? When in the process of their care? How do women react?

## View on talking to women PFD risk while pregnant/post partum generally

- What are your thoughts about discussing an individual woman’s PFD risk while she is pregnant and after the birth of her baby?
- What do you think women should be told about PFD?
- What in your view do women want to know about PFD and why?
- Are there things that get in the way of talking to women about PFD?

## Introduction to UR-CHOICE process

- Ask healthcare professional to look at the calculator and ask them if they have the data on the two fictional women How would you feel about completing this in practice with women?
- What impact do you think giving that information would have for your practice?
  - Benefits
  - Drawbacks
- What impact do you think giving that information would have for women?
  - Benefits
  - Drawbacks
- If a woman’s risk was higher than the general population what do you think you would advise? What are the implications for the woman? [probe around risk reduction; probe around thoughts on delivery method]
- When would be the best time to do this (if at all)
- Who do you think is best placed to discuss this information with women (if anyone)
  - Probe obstetrician
  - Probe midwife
  - Probe primary care
- Do you think knowing this kind of information would cause a women’s to be anxious?
- If you were to be asked to be a centre in a trial about the UR-CHOICE process – do you think that you would (hypothetically) be willing or not?

## Closure

Thank you for talking with me today. The interview we have recorded will be removed from the recording device as soon as possible and stored securely. When the interview is typed up (transcribed), all identifying information will be removed. We will study the information you have given us alongside that given by other participants. It will not be possible to identify you from the information given.

## Desire to have study results sent to him/her

Explore if healthcare professional would like to have a summary of the study results sent to him/her and if so if it is acceptable to send them to the email address used to reply.

**Thank you for taking part.**
